# Medical Students’ Perspectives on Careers in Hospital Medicine: A National Study

**DOI:** 10.15694/mep.2017.000207

**Published:** 2017-11-20

**Authors:** Anne Catherine Miller Cramer, Sandra A. Ham, Yassen Alkaddoumi, Kamel Ibrahim, Shalini T. Reddy, John D. Yoon

**Affiliations:** 1 The University of Chicago; 2 Mercy Hospital and Medical Center

**Keywords:** specialty choice, hospitalist, burnout, national survey, medical students

## Abstract

This article was migrated. The article was marked as recommended.

**Purpose:** Concerns over burnout and other factors may influence whether students pursue hospital medicine as a career. We investigate whether there are certain predictive factors that ultimately play a role in medical students’ career interest in hospital medicine.

**Methods:** In January 2011, 960 third-year medical students from 24 U.S. allopathic medical schools were surveyed at baseline, and six to nine months later when they became fourth-years at follow-up. Hospitalist-oriented students were categorized as those students who indicated interest in the specialties of family medicine, internal medicine, or pediatrics, and who indicated that they were “very likely” or “somewhat likely” to become a hospitalist. Respondents were also asked to respond to a list of seven factors that potentially influenced their specialty choice.

**Results:** Adjusted response rate for the fourth-year survey was 50% (n=463/919). Medical students considering hospitalist careers were more likely to report that perceived burnout between various specialties played an important influential role in their specialty decision-making (49.7% [42.2-57.2%], vs. non-hospitalists 39.9% [32.8-47.0%], P=0.03).

**Conclusions:** Given that students are reporting burnout as a factor in their decision-making in favor of hospitalist careers, further studies are needed to explore what aspects of a hospitalist career are appealing to students.

## Introduction

Hospital medicine has become a more pervasive specialty over the past 20 years, with many hospitals adopting hospitalist-based models of care. As this new care model has emerged, physician burnout has become a “hot-button issue” for the field of hospital medicine. ^1^ Burnout is prevalent in both trainees and a challenge for clinicians in hospital medicine nationwide. Interestingly, a study recently found that burnout among hospitalists exists regardless of practice model. ^2, 3^ In spite of this, hospitalists do report satisfaction with their work-life balance. ^4^ Medical student specialty choice is a complex issue, and one that has been studied extensively. However, factors that influence why students pursue hospital medicine as a career are less well studied. Our paper investigates whether there are certain predictive factors that ultimately play a role in medical students’ reported interest in pursuing hospital medicine as a long-term career, and whether the concern of burnout plays an important factor in the decision.

## Methods

The sample was drawn from the American Medical Association Physician Master File, using a systematic sampling plan with probability proportional to size and implicit stratification. We selected 24 of the U.S. allopathic medical schools, and then randomly selected 40 students from each school, to achieve a nationally representative sample. We mailed a confidential, self-administered, 12-page questionnaire to the stratified random sample of 960 third-year U.S. medical students in January 2011, with a follow-up survey in September 2011 when the third-year students became fourth year students. In order to make nationally representative estimates, case weights were employed to reflect sources of variance associated with the sample design and to adjust for potential nonresponse bias as described elsewhere. ^5^ We used descriptive statistics to summarize student demographics and predictor variables.

For specialty choice, a definition of “hospitalist” was provided to respondents on the survey, using the Society of Hospital Medicine (SHM) definition on the SHM website in 2011: *“A hospitalist is a physician whose focus is the general medical care of hospitalized patients.”* Hospitalist-oriented students were categorized as those students who indicated interest in the specialties of family medicine, internal medicine, or pediatrics, and who indicated that they were “very likely” or “somewhat likely” to become a hospitalist. All other responses were categorized as “non-hospitalist.” Respondents were also asked “How much do you think each of the following considerations will influence your specialty choice?” with a list of seven items ( Table 1). For each item, responses were dichotomized into *not influential* (“little to no influence” and “some influence”) vs. *influential* (“a lot of influence” and “the most possible influence”). We computed weighted estimates of percentages of students planning hospitalist vs. non-hospitalist careers. The association between becoming a hospitalist vs. not becoming a hospitalist and each of the items in Table 1 was assessed with a Chi square test. All analyses were performed using SAS 9.4 (Cary, NC, 2014).

Ethical approval: This study was approved by the University of Chicago Social Sciences Institutional Review Board in January 2011 (IRB #09-048).

## Results

After excluding 41 non-eligible respondents who were no longer students, the response rate for the first questionnaire was 61% (n=564/919) and 50% (n=463/919) for the follow-up questionnaire (total respondents without missing responses on survey items = 445). Women were more likely to report interest in careers as hospitalists (58.1% [49.3-66.8%], P=0.002). Higher levels (>$200,000) of student debt were negatively correlated with choosing hospital medicine (17.9% [9.2-26.7%], P=0.02). Race/ethnicity and immigration history were not associated with choosing hospitalist careers.

Table 1 shows which factors medical students perceive as important when choosing a career in hospital medicine. Significantly, medical students considering hospitalist careers were more likely to report that perceived burnout between various specialties played an important influential role in their specialty decision-making (49.7 [42.2-57.2], vs. non-hospitalists 39.9 [32.8-47.0], P=0.03).

**Table 1.** Factors that potentially influence specialty choice among U.S. fourth-year medical students, 2011

	Hospitalist (N=109) % (95% CI)	Not Hospitalist (N=336) % (95% CI)	Total % (95% CI)	P-value
Your financial debt at graduation				0.12
Important	12.1 (5.4-18.8)	18.9 (14.4-23.4)	17.2 (13.5-20.9)	
Not important	87.9 (81.2-94.6)	81.1 (76.6-85.6)	82.8 (79.1-86.5)	
Desire for a manageable lifestyle				0.29
Important	71.9 (62.2-81.6)	64.8 (57.8-71.9)	66.6 (61.3-71.8)	
Not important	28.1 (18.4-37.8)	35.2 (28.1-42.2)	33.4 (28.2-38.7)	
Family considerations and/or expectations				0.08
Important	57.7 (48.6-66.7)	48.8 (43.1-54.5)	51.0 (46.0-55.9)	
Not important	42.3 (33.3-51.4)	51.2 (45.5-56.9)	49.0 (44.1-54.0)	
Expected income for different specialties				0.17
Important	16.3 (9.6-23.0)	22.3 (17.2-27.4)	20.8 (16.7-25.0)	
Not important	83.7 (77.0-90.4)	77.7 (72.6-82.8)	79.2 (75.0-83.3)	
Desire to follow in the footsteps of a physician you admire				0.79
Important	21.3 (12.0-30.6)	22.7 (17.1-28.2)	22.3 (17.5-27.1)	
Not important	78.7 (69.4-88.0)	77.3 (71.8-82.9)	77.7 (72.9-82.5)	
A deep sense of calling to a particular specialty				0.61
Important	64.9 (52.8-77.1)	68.4 (62.8-74.1)	67.6 (63.0-72.2)	
Not important	35.1 (22.9-47.2)	31.6 (25.9-37.2)	32.4 (27.8-37.0)	
The extent to which physicians in different specialties seem to be burned out by their work				0.03
Important	49.7 (42.2-57.2)	39.9 (32.8-47.0)	42.3 (36.2-48.3)	
Not important	50.3 (42.8-57.8)	60.1 (53.0-67.2)	57.7 (51.7-63.8)	

## Discussion

Hospital medicine is a growing field and increasingly more attractive to graduating residents. However, increasing concerns of physician burnout are emerging. ^1- 4^ It would be valuable to be able to identify trainees at early stages of their careers to help them prepare for successful careers in hospital medicine. Our study looked to identify correlative factors among medical students who report interest in hospital medicine.

Several authors have previously investigated our question with conflicting results. Hauer et al ^6^ conducted a web-based cross-sectional survey of 1177 fourth-year medical students in 2007. These authors found that independent predictors of pursuing a career in general internal medicine included male gender, private medical school, favorable impressions of their internal medicine attending, favorable impressions of internists’ lifestyles, and favorable impressions about caring for internal medicine patients. ^6^ Similarly, a cross-sectional study ^7^ found that time with family was the factor most commonly reported as of high or very high importance to career decisions (69.6%). In that study, women were more likely to assign greatest importance to family time and long-term patient relationships. Across debt levels, financial considerations were of greatest importance more often for residents owing >$150,000. ^7^

Previous studies have shown that hospital medicine is an increasingly reported career choice for graduating residents in internal medicine (9.3% of graduating residents), through these studies suggest that many residents do not finalize this decision until their final year. ^8^ Looking at our results, a higher percentage of women select hospital medicine as a likely career choice. This is most likely related to a shorter length of training, as well as to perceived increased flexibility in scheduling. In addition, higher levels of expected student debt were negatively correlated with choosing hospital medicine as a career (data not shown). Table 1 supports this correlation, as medical students who assigned importance to the expected income of certain specialties were less likely to report interest in a career in hospital medicine. We also tested whether other factors were correlated with choosing careers in hospital medicine include a desire for a manageable lifestyle, family considerations, a sense of calling and the desire to follow in the footstep of an admired physician. We did not find statistically significant associations between these factors and the decision to choose a hospitalist career.

Perhaps most interestingly, medical students did report considering burnout as a factor in their decision-making in favor of hospitalist careers, perhaps reflecting a perception that careers in hospital medicine would help them avoid burnout in the future. Interestingly, when we actually assessed students’ experiences of burnout at the time of the survey (using a two-item validated short form of the Maslach Burnout Inventory), ^9^ burnout was not associated with specialty choice (data not shown). We hypothesize that their interactions with hospital medicine providers in the academic setting have perhaps exposed them to providers who are experiencing less burnout than their non-academic counterparts. Hospitalists are well-liked in the academic setting, with medical students reporting that hospitalists are more effective at delivering inpatient education than non-hospitalist general internists. ^10^ Gerber has previously described the “interpersonal coping mechanism of modeling” in which trainees identify a trusted clinical attending physician role model who can help them cope with conditions of high stress. ^11^ Hospitalist educators should, as role models and mentors, help students and resident trainees develop strategies for avoiding burnout, including resilience techniques. ^12^

This study has important limitations. Firstly, this was a post-hoc analysis of cross-sectional data from a larger study (Project on the Good Physician ^5^), so we could not definitively establish causality in our reported associations. Secondly, though we achieved a good response rate, non-respondents may differ from respondents in ways that bias our results. Lastly, the intentions of graduating medical students in some cases will not lead to actual practice in hospitalist careers for a variety of reasons not examined in this study.

In conclusion, our national survey of medical students found that medical students considering hospitalist careers were more likely to report that perceived burnout between various specialties played an important influential role in their specialty decision-making. Given that students are reporting that they are considering burnout as a factor in their decision-making in favor of hospitalist careers, further studies are needed to explore what aspects of a hospitalist career are appealing to students.

## Notes On Contributors

Dr. Cramer is Clinical Associate in the Section of Hospital Medicine at the University of Chicago.

Ms Ham is Senior Statistician at the Center for Health and Social Sciences at the University of Chicago.

Dr. Alkaddoumi is third-year internal medicine resident at Mercy Hospital and Medical Center.

Dr. Ibrahim is third-year internal medicine resident at Mercy Hospital and Medical Center.

Dr. Reddy is Professor of Medicine at the University of Chicago and Associate Program Director at Mercy Hospital and Medical Center.

Dr. Yoon is Assistant Professor of Medicine at the University of Chicago and Assistant Program Director at Mercy Hospital and Medical Center.
