# Supplementary material for: Medical Students’ Perspectives on Careers in Hospital Medicine: A National Study
Source: MedEdPublish (2016). 2017 Nov 20;6:207. [Version 1] doi: 10.15694/mep.2017.000207 (PMC10885226; doi:10.15694/mep.2017.000207)
Supplement: Peer review history

# Reviewer report: Reviewer response for version 1

**DOI:** 10.21956/mep.19395.r28441

**Published:** 2017-11-29

**Authors:** Neel Sharma

**Affiliations:** 1 Albert Einstein College of Medicine, Montefiore Medical Center

This review has been migrated. The reviewer awarded 5 stars out of 5 Physician burnout is certainly a hot topic - that seems a global issues. Each nation has reasons for such an occurrence which I witnessed during my sabbaticals. From a UK POV, the junior doctor strike highlighted discontent in view of increasing admissions, less resources, understaffing and so on. Public health systems and private bring their sources of burnout. Recognising burnout is essential and ensuring each individual chooses the career path suitable to them is also key. For myself that was a mix of clinical care and academic pursuits. Each individual should pursue their passion - initially this could lead to certain sacrifices (financial for example) but long term its a winning solution.

Reviewer Expertise:

NA

No decision status is available

# Reviewer report: Reviewer response for version 1

**DOI:** 10.21956/mep.19395.r28442

**Published:** 2017-11-21

**Authors:** BALAJI ARUMUGAM

**Affiliations:** 1 TAGORE MEDICAL COLLEGE AND HOSPITAL

This review has been migrated. The reviewer awarded 4 stars out of 5 The study was well conducted and the results are really quiet interesting. The author had explored the predictors for choosing hospitalist and non hospitalist. The study participants considering hospitalist careers were more likely to report that perceived burnout between various specialties played an important influential role in their specialty decision-making. The data seems to be little older and almost 6 years back. The perceptions might have been changed in the current scenario. In depth interview and qualitative studies will explore the real facts about choosing hospitalist and non hospitalist as career.

Reviewer Expertise:

NA

No decision status is available

# Reviewer report: Reviewer response for version 1

**DOI:** 10.21956/mep.19395.r28443

**Published:** 2017-11-20

**Authors:** Paula Whittaker

**Affiliations:** 1 University of Manchester

This review has been migrated. The reviewer awarded 4 stars out of 5 Really interesting method to get an insight into the factors influencing medical students career choice. Would be very useful for specialities that struggle to recruit, e.g. psychiatry in England in helping to frame career discussions with medical students.

Reviewer Expertise:

NA

No decision status is available
